# Predator arrival elicits differential dispersal, change in age structure and reproductive performance in a prey population

**DOI:** 10.1038/s41598-018-20333-0

**Published:** 2018-01-31

**Authors:** A. Payo-Payo, A. Sanz-Aguilar, M. Genovart, A. Bertolero, J. Piccardo, D. Camps, J. Ruiz-Olmo, D. Oro

**Affiliations:** 1 GEP, IMEDEA (CSIC-UIB), Esporles, Spain; 2 GEDA, IMEDEA (CSIC-UIB), Esporles, Spain; 3 Ecology Area, Dept. Applied Biology, Miguel Hernández University, Elche, Spain; 4 IMEDEA (CSIC-UIB), Esporles, Spain; 5 CEAB (CSIC), Blanes, Spain; 6 Associació Ornitològica Picampall de les Terres de l’Ebre, Amposta, Spain; 7 General Directorate for Environmental Policy, Ministry of Territory and Sustainability, Government of Catalonia, Barcelona, Spain; 8 General Directorate of the Natural Environment and Biodiversity, Ministry of Agriculture, Livestock, Fisheries, Food and Natural Environment, Government of Catalonia, Barcelona, Spain

## Abstract

Predators are an important ecological and evolutionary force shaping prey population dynamics. Ecologists have extensively assessed the lethal effects of invasive predators on prey populations. However, the role of non-lethal effects, such as physiological stress or behavioural responses like dispersal, has been comparatively overlooked and their potential population effects remain obscure. Over the last 23 years, we developed a mark-recapture program for the Audouin’s gull and an intensive carnivore monitoring program to assess how the appearance and invasion of the study site by carnivores affects population dynamics. We evaluate changes in turnover of discrete breeding patches within the colony, age structure and breeding performance. Once carnivores entered the colony, the number of occupied patches increased, indicating a higher patch turnover. Breeders responded by moving to areas less accessible to carnivores. More importantly, the presence of carnivores caused differential (and density-independent) breeding dispersal: experienced, better-performing breeders were more likely to leave the colony than younger breeders. This differential dispersal modified the age structure and reduced the reproductive performance of the population. Our results confirm the importance experience in the study of populations. The role of differential dispersal for animal population dynamics might be more important than previously thought, especially under scenarios of global change.

## Introduction

Biological invasions are one of the most important drivers of ecological change globally^1,2^. In the last few decades the devastating effects of carnivore introductions by humans — in previously carnivore-free areas — have been linked to the decline and/or the extirpation of numerous native animal populations worldwide^3,4^. This is particularly true for numerous “naïve” prey species — such as seabirds — that evolved in carnivore-free environments and exhibit ineffective antipredator responses to novel predators^3,5,6^. Lethal effects of invasive species have been thoroughly studied in wild animal populations^7,8^. However, such invasions are complex phenomena often involving both lethal and non-lethal effect^9^. In many cases native species populations — and individuals within them — respond to invasive species by adjusting their behaviour to survive and reproduce within the same areas and/or modifying their spatial distribution by dispersing^5,10^. Non-lethal effects of invasive predators, such as dispersal, have also been studied but mainly focusing on species site fidelity and distribution changes^11^.However, dispersing to a new environment can be risky. In fact, philopatry has evolved as an advantageous strategy for many species, which benefit from previous knowledge about their environment^4,12,13^. However, from an evolutionary point of view, vagility of some organisms (such as birds, bats or fish) should allow a response to predators presence, by dispersing to other patches and, therefore, avoiding the lethal effects of predation^14–16^. The presence of predators may create a *landscape of fear*, and behavioural responses, such as dispersal, should appear^3,4,14,17^.

It has been recently suggested that individuals may respond differently to environmental change depending on their previous experience, showing innovative behaviours, such as changes in migration patterns or the colonization of new environments^18,19^. The fitness of long-lived species usually increases with age, reaching its maximum value in intermediate ages as a result of increased experience and skill enhancement^20–22^. Therefore, experience-dependent behavioural responses can potentially result in shifts in the population age-structure and reproductive value modifying a population’s ability to respond to perturbations^23–25^.

Here, we explore potential non-lethal effects of invasive native mesocarnivores at a previously predator-free colony of Audouin’s gull (*Ichthyaetus audouinii*)^26^. This species usually lays 3 egg-clutches, it is endemic to the Mediterranean and until the 1980s its population was regarded as threatened^27^. Its main food source are fisheries discards and they can represent up to 75% of their diet during the rearing period^28^. Before the 1980’s the species was only known to breed in carnivore free islands in the Mediterranean. In 1980’s the species colonized Ebro Delta (40°34′39″N 0°35′42.7″E, Spain) — a mainland patch free of carnivores. Since then, the number of breeding individuals progressively increased until the mid 2000s, when population density began to decline at a steady rate^14,29^. Carnivores (i.e. foxes, *Vulpes vulpes*) were first spotted in the area in 1994, after entering the colony through El Trabucador sandbar, but their presence was only regular from 1997 onwards.

Undelying causes of recent decline in this Audouin’s gull population remain unclear. However, it seems to be linked to dispersal and the settlement of new colonies as a result of density dependence, appearance of carnivores and/or extreme weather events^14,19^. Since the species is vagile but exhibits philopatric behaviour we expect to find resilient mechanisms to cope with carnivores, such as changes in the spatial distribution of breeding patches within the colony^5,30,31^. Previous studies suggest that more experienced individuals are more competent in dealing with environmental change^18,19^ and show higher breeding performance^24^. We hypothesize that the occurrence of carnivores stimulates experienced, but not inexperienced, birds to undergo breeding dispersal, which causes an increase in the proportion of younger and inexperienced breeders occupying habitats with carnivores. This increase in the proportion of younger individuals should then have effects on breeding performance, potentially influencing egg volume, clutch size and/or breeding success.

## Results

**Figure 1:**
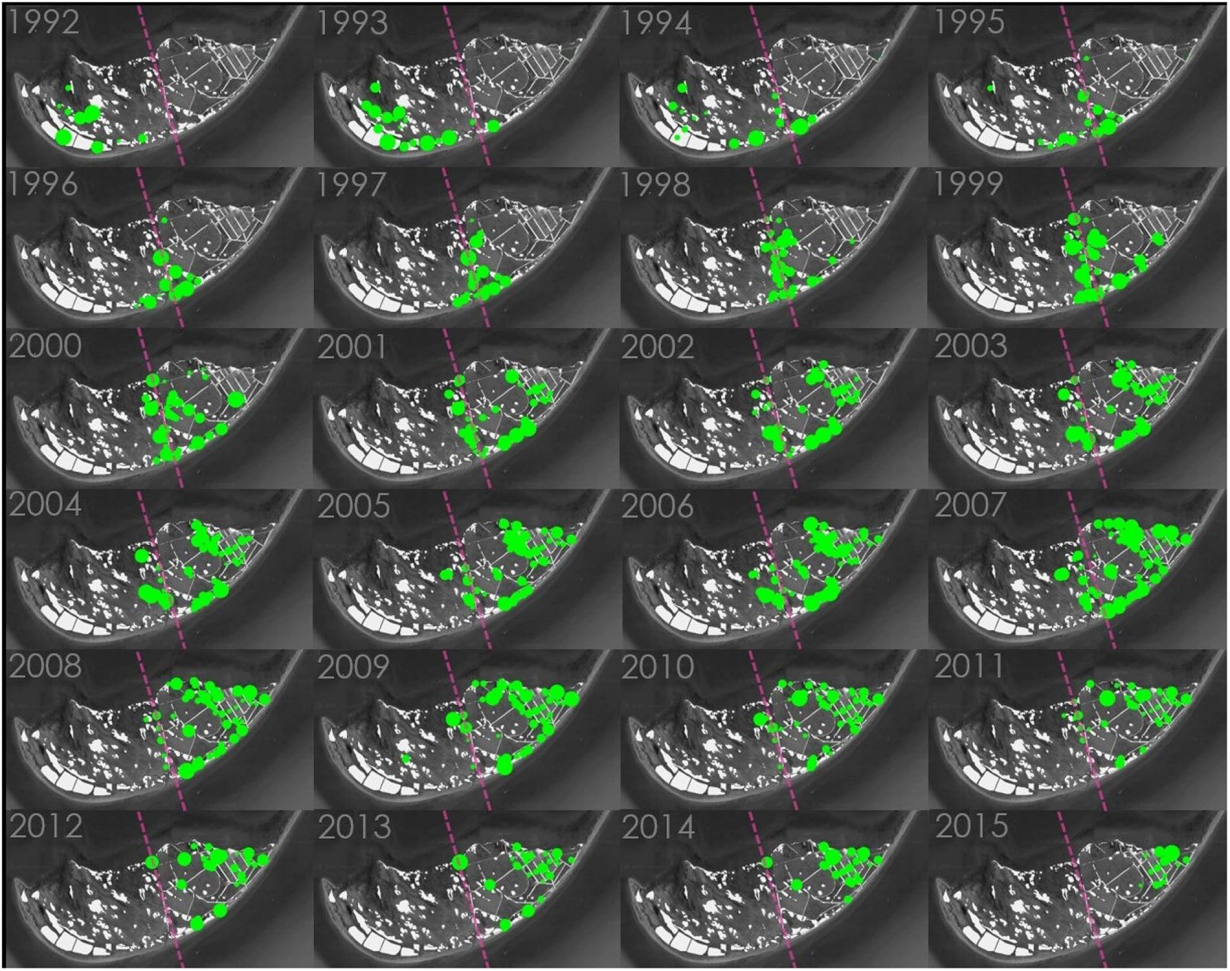
Spatiotemporal distribution of Audouin’s gull breeding patches at the Ebro Delta colony. White polygons represent the spatial distribution of potentially suitable breeding areas. Each annual image covers 60 km^2^. Green circles represent approximate locations of occupied patches and circle size is proportional to the number of individuals breeding in each patch. Predators were regularly present in the colony since 1997. Purple dashed lines represent the interphase between unmanaged dune vegetation (west) and human managed salt pans (east). Maps modified from: Google, 2016 DigitalGlobe.

**Figure 2:**
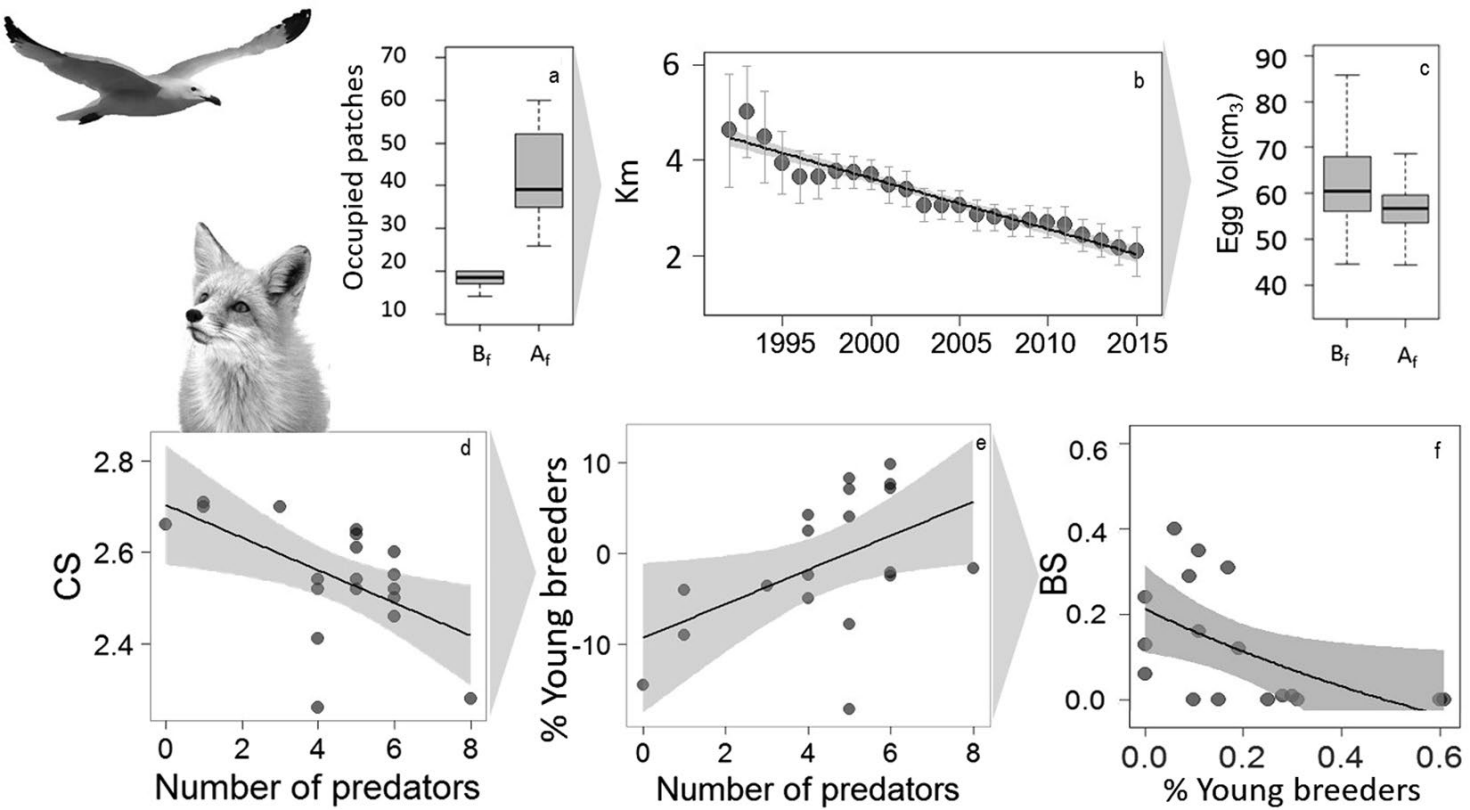
Non-lethal effects of predator presence in the Audouin’s gull breeding colony (Ebro Delta, Spain). (**a**) Changes in number of occupied patches before, B_f_, and after, A_f_, predators entered the colony. (**b**) Temporal evolution (1992-2015) of the mean distance (±1.96 SE) between the breeding area centroid and the eastermost point of the Ebro Delta colony (40.59 N, 0.71E). (**c**) Egg volume, Egg Vol before, B_f_, and after, A_f_, predators entered the colony. (**d**) Mean clutch size, CS and (**e**) proportion of young and inexperienced breeders in relation to the number of predators (relative difference between the observed and expected young and experienced breeders, positive numbers indicate lower proportions of experienced breeders as expected). Relationship between breeding success and % of young breeders, example from the year 2012 (**f**). Audouin gull modified from authors own photo. Fox modified from photo “Redfox10”. ©Cadigan. 2014: https://flic.kr/p/kFvEbZ. The images can be used under a CC by 2.0 https://creativecommons.org/licenses/by/2.0/.

**Figure 3:**
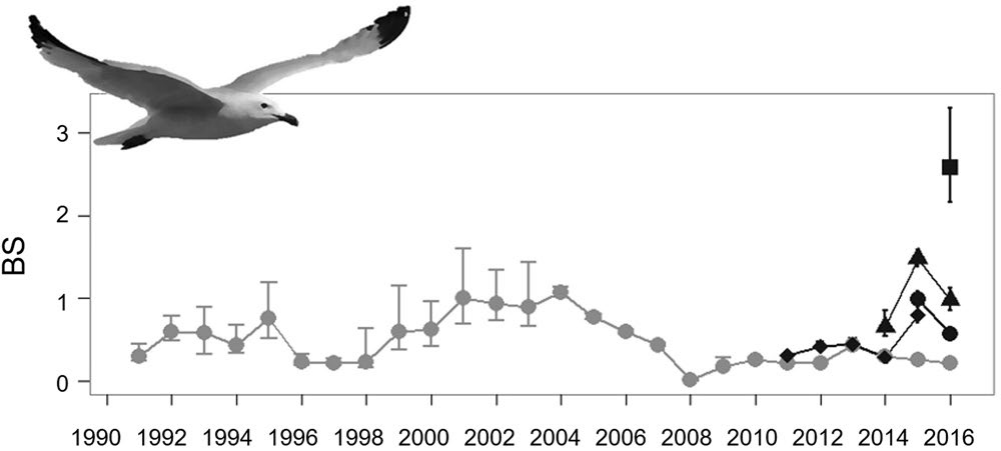
Breeding success (chicks/pair) and 95% IC of different Audouin’s gull breeding colonies: Punta de la Banya (light grey circles), La Ràpita (diamonds), Sant Antoni (solid dots), Tarragona Port (square) and Castellón (triangles). Audouin gull modified from authors own photo. The image can be used under a CC by 2.0 https://creativecommons.org/licenses/by/2.0/.

The presence of carnivores correlates with increased rates of patch occupation (N, Figs 1, 2a). Breeders responded to carnivore presence by moving eastwards (~180 m/y, R^2^ = 0.79, p-value < 0.001) towards patches surrounded by water, and therefore less accessible to carnivores (Fig. 1 in the main text and Tables 1, 2 in electronic Supplementary Material S1 and video in electronic Supplementary Material S2). Neither the presence nor abundance of carnivores triggered immediate colonization or extinction of patches (see electronic Supplementary Material S1, Table 1). The difference between the expected and the observed proportion of young and inexperienced breeders was positively correlated with the number of carnivores present at the colony (R^2^ = 0.33, F_1,21_ = 10.55, p-value = 0.004). Egg volume was lower after carnivores entered the study area (Fig. 2c and electronic Supplementary Material S1, Tables 1–2). Clutch size was negatively correlated with the number of carnivores (Fig. 2d and see electronic Supplementary Material S1, Tables 1–2). Egg volume, clutch size and breeding success in the different patches in 2012 was negatively correlated with the proportion of young and inexperienced breeders in each of the patches, however this relationship was only significant for breeding success (Fig. 2f and see electronic Supplementary Material S1, Tables 1–2). Breeding success decreased during the study period (Fig. 3, R^2^ = 0.48, F_1,15_ = 15.64, p-value = 0.001) and it was lower at the Ebro Delta colony than in any other breeding colony with no carnivores (Fig. 3).

## Discussion

Our results suggest that the effects of density-independent perturbations, such as the invasion by carnivores, can drive changes in population age structure and consequently, in reproductive value. Moreover, our results confirm previous evidence indicating that the underlying mechanism driving the spatial scale of dispersal is experience^19^. Non-lethal effects — such as behavioural responses — seem to be more important than previously thought for population dynamics and may have key implications for population resilience.

Philopatry is a common phenomenon among vertebrates and many species benefit from familiarity with the environment when conditions are relatively predictable^13,32^. However, when environmental variability increases, behavioural responses such as breeding dispersal are crucial to an effective response to sudden environmental changes^19,33,34^. Previous studies in the same area reporteda large scale non-random natal and breeding dispersal process (10–100 km) resulting in the occupation of carnivore-free patches and severe population consequences^14,19^. Here, we also identified shifts in the breeding area distribution at a small spatial-temporal scale (<1 km/y). Our results are in agreement with other studies indicating that bird distribution is conditioned by the presence of carnivores^35^. Patch occupation turnover within the studied area resulted in the displacement of breeding patches towards more anthropic but less accessible to carnivores breeding area^36^. Patch occupation turnover is likely a resilient mechanism to face mesocarnivore presence and to avoid the inherent risks of long-distance dispersal^19,37^. Previous studies in the same colony showed that some of those patches with lower mesocarnivore accessibility experienced higher breeding success^19^. However, breeding success decreased during the study period not only in patches with higher mesocarnivore accessibility but overall in the studied colony. Several possible and nonexclusive hypotheses have been proposed to explain changes in breeding success across taxa. First, low food availability, poor parental condition and high density-dependence during the breeding period are usually linked to decreases in individual and population breeding success^38,39^. Second, behaviors that are associated with predation risk, including vigilance, alarm, avoidance, escape and defence can reduce breeding success through increased physiological stress, reduced foraging efficiency or diminished parental care^4^. Third, populations experiencing the presence of predators can also experience reduced breeding success through direct brood consumption or predation on breeding adults^40–42^.

Here, we propose a density-independent mechanism explaining the decrease in the overall population’s breeding success. Our results suggest that the presence of predators drives changes in population age structure, decreasing proportions of experienced breeders and therefore, reducing the population breeding performance in the study area and increasing fitness in other breeding colonies outside of the study area. Fitness of long-lived species usually increases with age and breeding experience, reaching its maximum value at intermediate ages^20,23,24^. Previous studies of the species at the study area revealed that site fidelity was the most common strategy among breeders. However, breeding sites can be abandoned after severe environmental change, such as entrance of predators or changes in habitat quality^14,31,42^. Drivers underlying decision rules about dispersal are often complex and involve non-linear dynamics^14,19^. However, it has been recently discovered that the establishment of new sites and innovative responses to environmental change can be driven by age or experience^18,43^.

For instance, crane groups containing experienced members, established new overwintering sites closer to the breeding grounds^18^. Moreover, Audouin’s gulls stablishing new colonies around the study area were also experienced breeders^19^. Therefore, we suggest that large-scale non-random dispersal might be the causes of the observed changes in the age structure at the source population. This may also be linked to another underlying mechanism, namely relaxed competition for limited breeding sites would also favour recruitment of competitively inferior individuals (i.e., young and inexperienced breeders). Therefore, we suggest that perturbations can also drive changes in the breeding performance of the population through the selection of young and inexperienced breeders with lower breeding success^20,24^. Moreover, dispersal and philopatry seem to result in non-linear relationships between the colonisation of new patches and adverse breeding conditions such as the presence of predators. This seems to agree with previous findings: experience and aging involves the enhancement of individual quality through the improvement of ecological knowledge. Therefore, experienced breeders act as an information repository, which is extremely valuable when making risk-taking decisions in the face of environmental change^18,19^.

In summary, we show that non-lethal effects of a carnivore invasion, such as non-random dispersal, carry large population trade-offs (i.e. severe decrease in breeding success and population size) that may be comparable to lethal effects themselves^44^. Experience seems to be a valuable resource to overcome uncertainty when dispersing and appears to drive the gradient between small and large scale dispersal. Our work emphasizes that it is not safe to assume that behavioural responses have no or limited effects on population dynamics and that such effects should be accounted for in population modelling. We also emphasize the importance of carefully considering the role of non-lethal effects and experience, especially under scenarios of global change that predict increases in magnitude and the frequency of extreme events, biological invasions and species distribution shifts.

## Methods

### Species and the study area

The Audouin’s gull is an endemic Mediterranean seabird^45^. The species colonized the Ebro Delta in 1981 (40°37′N, 00°35′E). This area has been annually monitored and a mark-capture-recapture program is ongoing, with more than 33.000 individuals marked. Until the mid-2000s, the colony hosted 70% of the species’ world population. However, since 2002 the Ebro Delta population has declined^14,19^.

Breeding individuals in the colony are spatially aggregated in discrete dunes and dikes (hereafter patches)^46^. Patch size is very variable; it ranges from 1 to 3400 breeding pairs concentrated in aggregations (around 30 cm is the minimum distance between nests). Separation distance between patches is greater to the average distance between nests (minimum distance 15 m). Patches located at the eastern part of the colony — human managed saltpans — are usually surrounded by water and, therefore, more protected from carnivores, while those located at the western part of the colony — unmanaged dune vegetation — are mostly not.

The presence of mesocarnivores was first reported in 1994, and since 1997 they have been regularly present at the breeding colony. Wildlife managers and staff perform annual systematic surveys to detect carnivores (mostly foxes and badgers, but occasionally otters, stone martens, least weasels, cats and dogs). The study area was sampled for a minimum of 48 days each year, adding all opportunistic observations made throughout the season by the staff (daily presence of >8 h). The study area is a peninsula of limited dimensions (9.0 × 4.0 km) connected with the mainland by a narrow sand bar (El Trabucador, 6.0 × 0.1 km). The habitat is open and vegetation is sparse, sandy and muddy soils facilitate footprint detection. Moreover, used or abandoned dens are systematically checked for carnivore presence and evidences of predation such as prey remains. Recorded information included: location, number of individuals, number of family units and reproductive success (puppies’ footprints and accumulations of prey in the burrows in the case of fox and badger). Given the intensity of our surveys and the characteristics of the study area, we can assume that carnivore detection was high, and provides a robust estimate of the minimum number of unique predators in the study area.

We used this information to define two different indicators of mesocarnivore activity: first, a presence measure distinguishing before (<1997) and after (≥1997) predators were present; and second, an abundance measure accounting for the number of mesocarnivores present in the study area in a given year. Foxes represent 90% of the mesocarnivores present in the study area. Foxes are generalist species^47^ that, during the breeding season, act as specialist predators on waterbird species in the study area, where other prey are almost absent. These scales of resolution allowed us to assess the effects of carnivore presence on three different population traits: spatio-temporal distribution of breeding patches, age structure and breeding performance of Audouin’s gulls.

#### Spatio-temporal distribution of breeding patches

Patch occupation is registered every year, allowing us to record the number of occupied patches (N) and estimate the rate of occupation of new patches (Ncol) and rate of patch extinctions (Next). Ncol is the number of active colonies the same year and Next is the number of patches extinct from those patches occupied the previous year. None of the three variables were correlated with each other (R^2^ < 0.15). For each one of the three variables (N, Ncol, Next) and by means of a GLM we compared constant model (i.e. no effects) against two models including a presence/absence effect and an abundance effect. Presence and/or abundance of carnivores in the colony had an effect on any of the three variables. In addition to this, we assessed the effects of carnivore presence on the distribution of breeding patches in two different ways. First, we plotted a map documenting temporal changes in the spatial distribution of occupied patches across the entire study period (Fig. 1). Second, by means of GLMs, we quantitatively assessed if there was a shift in the breeding area distribution through the study period. We tested two alternative hypotheses, no shift (constant model) vs temporal effect (temporal model). The shift in the species breeding area distribution was assessed as the temporal change in the distance of the breeding area centroid to the easternmost point of the breeding colony (40°35′24″N, 0°42′39″). The breeding area centroid was estimated as the mean position of breeding patches weighted by the number of pairs breeding in each patch each year.

We selected the best model according to the Akaike information criteria (AIC; Burnham and Anderson, 2002). Models with Δ AIC < 2 were consider equivalent.

#### Age structure

Audouin’s gulls usually recruit at the age of 3 or 4 years and we assumed full recruitment at age 6^48^. Since age is a good indicator of breeding experience, we considered individuals between 3–5 years old as young and inexperienced breeders (YB). Determining the age structure of the population is a difficult task because age in this gull species cannot be assessed morphologically when individuals are 3 or more years old. Moreover, the proportion of live marked individuals of different ages depends on the numbers of fledglings marked by cohort and their subsequent survival^25^. showed that Audouin’s survival probability in our study area improves with age and that recapture probability does not depend on age. Therefore, we assumed that individuals experienced age dependent survival and that the recapture of breeders within the study area is age independent^25^. Under these assumptions, we evaluated the existence of temporal changes in the difference between the observed and expected proportion of young and inexperienced individuals (3–5 years old) as proxy of changes in age structure (CDIF_t_). We used a total of 14113 resightings of marked known-age gulls during the breeding period (April-June) to calculate the annual number of observed individuals of each age class. The annual number of expected individuals of age “a” at time “t” was estimated following equation (1). For instance, the expected number for individuals of age 3 at time 4 (N_3,4_) would be the number of chicks tagged at t = 1 (M_1_) that survived to t = 4 (N_3,4_ = M_1_Ф_1_Ф_2_Ф_3_), with Ф_a_ being the survival probability at age *a*. We calculated the annual difference in proportions of observed and expected young and inexperienced breeders (CDIFt, see electronic Supplementary Material S2, Fig. 4) following equation (2) (i.e., dividing the sum of young and inexperienced breeders,$\,\sum _{a=3}^{a=5}{N}_{a,t}$, by the total number of breeders, $\sum _{a=3}^{a=23}{N}_{a,t}$). For instance, the proportion of young and inexperienced breeders (3-5 years old) for time 4 would then be (N_3,4_ + N_4,4_ + N_5,4_)/N_3-23,4_. Finally, we calculated the annual difference between the observed and the expected annual proportions of young and inexperienced breeders as a proxy of population age structure, equation (3). Positive ${{\rm{CDIF}}}_{t}$ values will indicate an excess in the proportion of young and inexperienced breeders (or equivalently a lack of experienced breeders) compared to what we expected under the assumptions of our study.

1 $${N}_{a,t}={M}_{t-a}\prod _{a=1}^{a=n}{{\rm{\Phi }}}_{a}$$

2 $$Y{B}_{t}=\frac{{\sum }_{a=3}^{a=5}{N}_{a,t}}{{\sum }_{a=3}^{a=23}{N}_{a,t}}$$

3 $${{\rm{CDIF}}}_{t}=YB{O}_{t}-YB{E}_{t}$$

Finally, we tested the effect of the annual number of carnivores on age structure by means of comparing a constant model with a lineal regression model (LM) including the effect of the annual number of carnivores.

### Breeding performance

We tested for effects of absence/presence and intensity of carnivore presence on egg volume (Egg Vol), clutch size (CS) and breeding success (BS) between 1992 and 2015. Detailed information on Egg Vol, CS and BS estimations can be found in^19^. We used linear models to test effects on CS and BS, and general linear models including nest identity as a random factor to test Egg Vol differences. Finally, previous studies in the same study area showed that higher proportions of younger breeders in a given patch resulted in lower breeding performance^24,46^. Here, we show an example of the linear relationship between patch egg volume, clutch size and breeding success and age structure of all patches in a given year (2012). We also collected data on breeding success of newly formed colonies for a qualitative comparison with our study colony (Sant Antoni 40°43′22.81″N, 0°52′11.78″E; La Ràpita 40°37′0.50″N, 0°36′19.29″E, Tarragona Port 41°5′52.7″N 1°13′23.22″E and, Castellón, 39°57′49.40″N, 0°0′34.98″E). These colonies are located 10.4 km, 6.4 km, 74 km and 86 km from our study area^19^.

Analyses were implemented in R software and models were selected using the Akaike Information Criterion (AIC)^49^. Further details of data collection for each of the variables are in see electronic supplementary material S3.

### Ethics statement

This study complies with the European laws regulating research on animals. Spanish regulation does not require specific ethical approval for wildlife monitoring other than regular permits. Permits were granted by the Spanish Government: SF/134, SF/043, SF/097 and SF/269.

## Electronic supplementary material

video supplementary material

supplementary material

## References

[1] Clavero M, García-Berthou E (2005). Invasive species are a leading cause of animal extinctions. Trends Ecol. Evol..

[2] Lowry E (2012). Biological invasions: a field synopsis, systematic review, and database of the literature. Ecol. Evol..

[3] Wanless RM, Angel A, Cuthbert RJ, Hilton GM, Ryan PG (2007). Can predation by invasive mice drive seabird extinctions?. Biol. Lett..

[4] Cresswell W (2008). Non-lethal effects of predation in birds. Ibis.

[5] Igual, J. M., Forero, M. G., Gómez, T. & Oro, D. Can an introduced predator trigger an evolutionary trap in a colonial seabird? *Biol. Conserv*. **137** (2007).

[6] Sih A (2010). Predator–prey naïveté, antipredator behavior, and the ecology of predator invasions. Oikos.

[7] Croxall JP (2012). Seabird conservation status, threats and priority actions: a global assessment. Bird Conserv. Int..

[8] Lewison RL, Crowder LB, Read AJ, Freeman SA (2004). Understanding impacts of fisheries bycatch on marine megafauna. Trends Ecol. Evol..

[9] Owens IPF, Bennett PM (2000). Ecological basis of extinction risk in birds: Habitat loss versus human persecution and introduced predators. Proc. Natl. Acad. Sci..

[10] Stankowich T, Blumstein DT (2005). Fear in animals: a meta-analysis and review of risk assessment. Proc. R. Soc. B Biol. Sci.

[11] Hoover JP (2003). Decision Rules for Site Fidelity in a Migratory Bird, the Prothonotary Warbler. Ecology.

[12] Milot E, Weimerskirch H, Bernatchez L (2008). The seabird paradox: dispersal, genetic structure and population dynamics in a highly mobile, but philopatric albatross species. Mol. Ecol..

[13] Greenwood PJ, Harvey PH (1982). The Natal and Breeding Dispersal of Birds. Annu. Rev. Ecol. Syst..

[14] Fernández-Chacón A (2013). When to stay, when to disperse and where to go: survival and dispersal patterns in a spatially structured seabird population. Ecography.

[15] Munsch SH, Cordell JR, Toft JD (2016). Fine-scale habitat use and behavior of a nearshore fish community: nursery functions, predation avoidance, and spatiotemporal habitat partitioning. Mar. Ecol. Prog. Ser..

[16] Mikula P, Morelli F, Lučan RK, Jones DN, Tryjanowski P (2016). Bats as prey of diurnal birds: a global perspective. Mammal Rev..

[17] Laundré JW, Hernández L, Altendorf KB (2001). Wolves, elk, and bison: reestablishing the ‘landscape of fear’ in Yellowstone National Park, USA. Can. J. Zool..

[18] Teitelbaum CS (2016). Experience drives innovation of new migration patterns of whooping cranes in response to global change. Nat. Commun..

[19] Payo-Payo, A. *et al*. Colonisation in social species: the importance of breeding experience for dispersal in overcoming information barriers. *Sci. Rep*. **7** (2017). 10.1038/srep42866 PMC5314353 28211483

[20] Hernández, N., Oro, D. & Sanz-Aguilar, A. Environmental conditions, age, and senescence differentially influence survival and reproduction in the Storm Petrel. *J. Ornithol*. 1–11, 10.1007/s10336-016-1367-x (2016).

[21] Sergio F (2011). Variation in age-structured vital rates of a long-lived raptor: Implications for population growth. Basic Appl. Ecol..

[22] Sergio F (2014). Individual improvements and selective mortality shape lifelong migratory performance. Nature.

[23] Sanz-Aguilar A, Tavecchia G, Pradel R, Mínguez E, Oro D (2008). The cost of reproduction and experience-dependent vital rates in small petrel. Ecology.

[24] Oro D, Hernández N, Jover L, Genovart M (2014). From recruitment to senescence: food shapes the age-dependent pattern of breeding performance in a long-lived bird. Ecology.

[25] Payo-Payo, A., Genovart, M., Bertolero, A., Pradel, R. & Oro, D. Consecutive cohort effects driven by density-dependence and climate influence early-life survival in a long-lived bird. *Proc. R. Soc. B-Biol. Sci*. **283** (2016). 10.1098/rspb.2015.3042 PMC4855379 27122556

[26] BirdLife International. *Larus audouinii. The IUCN Red List of Threatened Species* (2015).

[27] Cramp, S. & Simmons, K. L. *The birds of Western Palearctic*. III, (Oxford University Press, 1985).

[28] Oro D, Ruiz X (1997). Exploitation of trawler discards by breeding seabirds in the north-western Mediterranean: differences between the Ebro Delta and the Balearic Islands areas. ICES J. Mar. Sci. J. Cons..

[29] Tavecchia G, Pradel R, Genovart M, Oro D (2007). Density-dependent parameters and demographic equilibrium in open populations. Oikos.

[30] Oro D, Tavecchia G, Genovart M (2011). Comparing demographic parameters for philopatric and immigrant individuals in a long-lived bird adapted to unstable habitats. Oecologia.

[31] Cam E, Oro D, Pradel R, Jimenez J (2004). Assessment of hypotheses about dispersal in a long-lived seabird using multistate capture–recapture models. J. Anim. Ecol..

[32] Greenwood PJ (1980). Mating systems, philopatry and dispersal in birds and mammals. Anim. Behav..

[33] Slatkin M (1987). Gene flow and the geographic structure of natural populations. Science.

[34] Gilpin, M. *Metapopulation Dynamics: Empirical and Theoretical Investigations*. (Academic Press, 2012).

[35] Tryjanowski P, Goldyn B, Surmacki A (2002). Influence of the red fox (Vulpes vulpes, Linnaeus 1758) on the distribution and number of breeding birds in an intensively used farmland. Ecol. Res..

[36] Barros, Á., Romero, R., Munilla, I., Pérez, C. & Velando, A. Behavioural plasticity in nest-site selection of a colonial seabird in response to an invasive carnivore. *Biol. Invasions* **18**, 3149–3161 (2016).

[37] Oro D (2009). Interference competition in a threatened seabird community: A paradox for a successful conservation. Biol. Conserv..

[38] Chastel O, Weimerskirch H, Jouventin P (1995). Influence of Body Condition on Reproductive Decision and Reproductive Success in the Blue Petrel. The Auk.

[39] Furness RW (2015). Density dependence in seabirds: Great Skuas Stercorarius skua start to breed at a younger age when conditions are better. Ringing Migr..

[40] Oro, D. A’s G. In *The birds of western Paleartic* (Ogilvie, M. A., 1998).

[41] Martínez-Abraín A, González-Solís J, Pedrocchi V, Genovart M, Oro D (2003). Kleptoparasitism, disturbance and predation of yellow-legged gulls on Audouin’s gulls in three colonies of the western Mediterranean. Sci. Mar..

[42] Libois E (2012). Nest boxes: A successful management tool for the conservation of an endangered seabird. Biol. Conserv..

[43] Sanz-Aguilar A, Jovani R, Melián CJ, Pradel R, Tella JL (2015). Multi-event capture–recapture analysis reveals individual foraging specialization in a generalist species. Ecology.

[44] Creel S, Christianson D (2008). Relationships between direct predation and risk effects. Trends Ecol. Evol..

[45] Oro D, Ruxton GD (2001). The formation and growth of seabird colonies: Audouin’s gull as a case study. J. Anim. Ecol..

[46] Genovart M, Jover L, Ruiz X, Oro D (2003). Offspring sex ratio in subcolonies of Audouin’s gull, Larus audouinii with differential breeding performance. Can. J. Zool..

[47] Ciampalini B, Lovari S (1984). Food habits and trophic niche overlap of the Badger (Meles meles L.) and the Red fox (Vulpes vulpes L.) in a Mediterranean coastal area. Z. Fíir Sáugetierkunde.

[48] Oro D, Pradel R (2000). Determinants of local recruitment in a growing colony of Audouin’s gull. J. Anim. Ecol..

[49] Burnham, K. P. & Anderson, D. R. *Model Selection and Multimodel Inference - A Practical Information-Theoretic Approach*. (Springer, 2002).

