# Supplementary material for: Predator arrival elicits differential dispersal, change in age structure and reproductive performance in a prey population
Source: Sci Rep. 2018 Jan 31;8:1971. doi: 10.1038/s41598-018-20333-0 (PMC5792507; doi:10.1038/s41598-018-20333-0)
Supplement: Supplementary file 2 — supplementary material [file 41598_2018_20333_MOESM2_ESM.pdf]

Predator arrival elicits differential dispersal, change in age structure and reproductive performance in a prey population.

Payo-Payo, A.[1]; Sanz-Aguilar, A.[1,2]; Genovart, M.[1,3]; Bertolero, A.[4]; Piccardo, J.[4]; Camps, D[5].; Ruiz-Olmo, J.[6]; Oro, D[1,3].

Electronic supplementary material

Electronic supplementary material S1 Model selection and model parameter tables.

Table 1. Model selection table for breeding parameters of Audouin's gulls breeding in the Ebro Delta. DF, degrees of freedom; DEV, deviance; AIC, Akaike's information criterion; ΔAIC, AIC difference with the best model; time (T); distance to the easternmost point of the breeding colony (D), clutch size (CS), egg volume (EGG VOL), rate of patch extinctions (N_{EXT}), rate of patch colonizations (N_{COL}), number of occupied patches (N).

Model	DF	DEV	AIC	ΔAIC	P-value
D NO EFFECT	1	45.24	87	38.00	
D T	2	8.66	49	0.00	***
N_{EXT} NO EFFECT	20		148.07	0.00	
N_{EXT} PRESENCE	19	0.00	150.07	2.00	
N_{EXT} ABUNDANCE	19	0.24	149.83	1.76	
N_{COL} NO EFFECT	21		131.90	0.00	
N_{COL} PRESENCE	20	1.03	132.87	0.97	
N_{COL} ABUNDANCE	20	-0.16	133.03	1.13	
N NO EFFECT	22		240.92	65.03	
N PRESENCE	21	67.03	175.89	0.00	***
N ABUNDANCE	21	-8.49	184.38	8.49	
EGG VOL NO EFFECT	4	257694	259759	41.00	
EGG VOL PRESENCE	5	257651	258377	0.00	***
EGG VOL ABUNDANCE	5	257681	259271	30.00	
CS NO EFFECT	23		-18.77	2.01	
CS PRESENCE	22	-0.07	-17.29	2.49	
CS ABUNDANCE	22	-0.08	-20.78	0.00	***

Table 2: Results from Linear Mixed and General Linear Modelling. Intercept (i) and slopes (β) for the fixed variables: predator presence (β_{after}), predator number (β_{Npred}), year (β_{year}); and the random variables: nest and year ($\beta_{residual}$), effects on clutch size (CS), egg volume (EGG VOL), distance(D), number of occupied patches(N) Est, estimate; SE, standard error; Var, variance; SD, standard deviation. Models are noted as in Table 1.

	D		N		CS		EGG VOL	
Fixed	Est	SE	Est	SE	Est	SE	Est	SE
i	360.99	37.07	2.99	0.09	-9.89	2.68	63.67	0.59
β_{after}			0.75	0.09			-6.9	0.63
β_{Npred}					1.98	0.61		
β_{year}	-0.17	0.02						
Random							Var	SD
i_{nest}							8.35	2.89
i_{year}							0.93	0.97
$\beta_{residual}$							13.64	3.69

Electronic supplementary material S2: Extended results.

Video 1: Temporal evolution (1992-2015) of Audouin's gull breeding patches at the Ebro Delta breeding colony. Circle size is proportional to the number of pairs breeding. Maps were built in R Software [7]. Maps copyright: 2016 © Google, DigitalGlobe.

Pach turn-over Ebro Delta 1992-2015.mp4

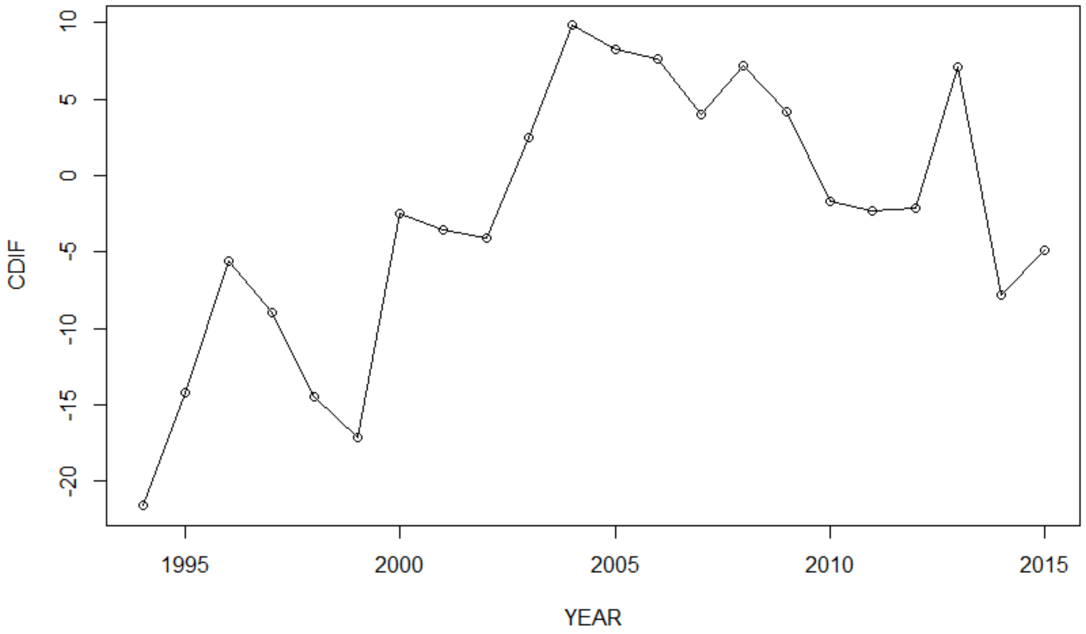

Figure 4. Annual difference in proportions of expected and observed young and inexperienced breeders (YB) at Ebro Delta Colony between 1993 and 2015.

References:

7. R Core Team. *R: A language and environment for statistical computing.* (R Foundation for Statistical Computing, 2013).

Electronic supplementary material S3: Extended methodology.

Predator presence surveys

Natural park staff performs systematic diurnal and nocturnal walks to detect tracks and locate and georeference the burrows of potential predators throughout the breeding season (Fig. 1. Foxes,

Vulpes vulpes, badgers, *Meles meles*; Beech marten, *Martes foina*; least weasel, *Mustela nivalis*; otters, *Lutra lutra*; cats, *Felis catus*; dogs, *Canis familiaris*).

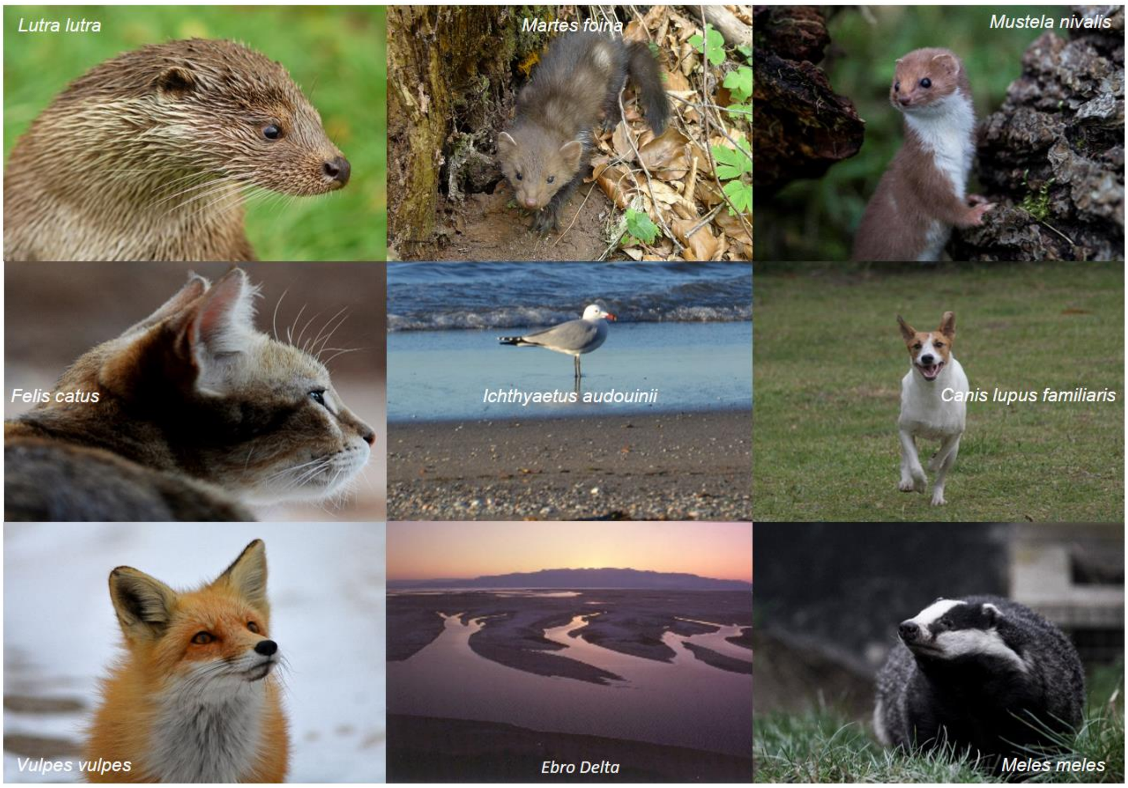

Figure 1. Habitat of the Audouin's gull (*Ichthyaetus audouinii*) breeding colony at Ebro Delta and potential predators reported at the Ebro Delta colony: otters (*Lutra lutra),* beech marten (*Martes foina),* least weasel (*Mustela nivalis),* cat (*Felis catus),* dog (*Canis familiaris),* foxes (*Vulpes vulpes)* and badgers (*Meles meles*). Images were modified from Flickr photos: "Audouin's Gull." © Birdwatching BCN 2013: https://flic.kr/p/e22P1e. "Red fox 10" © Cadigan. 2014 https://goo.gl/UwZvHz. "Badger" © Blg ashb. 2015; https://flic.kr/p/r2bEQV. Breech marten © Steinmarder. 2012: https://flic.kr/p/cD4bry. "Least weasel" © Blg-ashb. 2015: https://flic.kr/p/qYTkSb. "Otter". Trimming 2010: https://flic.kr/p/8Qh6XN. "Cat" © Kamaljith. 2011: https://flic.kr/p/9hGdy5. Dog. © Cole. 2012: https://flic.kr/p/brLXug. Ebro Delta © Oro 2004 All images are licensed under a CC BY 2.0 license https://creativecommons.org/licenses/by/2.0/.

Moreover, the colony occupies a relatively small sandy peninsula (Punta de la Banya, Ebro Delta, 2500 ha, Figure 2.a-d) characterized by compacted sandy substrate with low and patchy halophyte vegetation, which makes predator detection easy. We used the number of carnivores detected annually by the Ebro Delta Natural Park Environmental Agency during systematic surveys as a proxy of predator abundance.

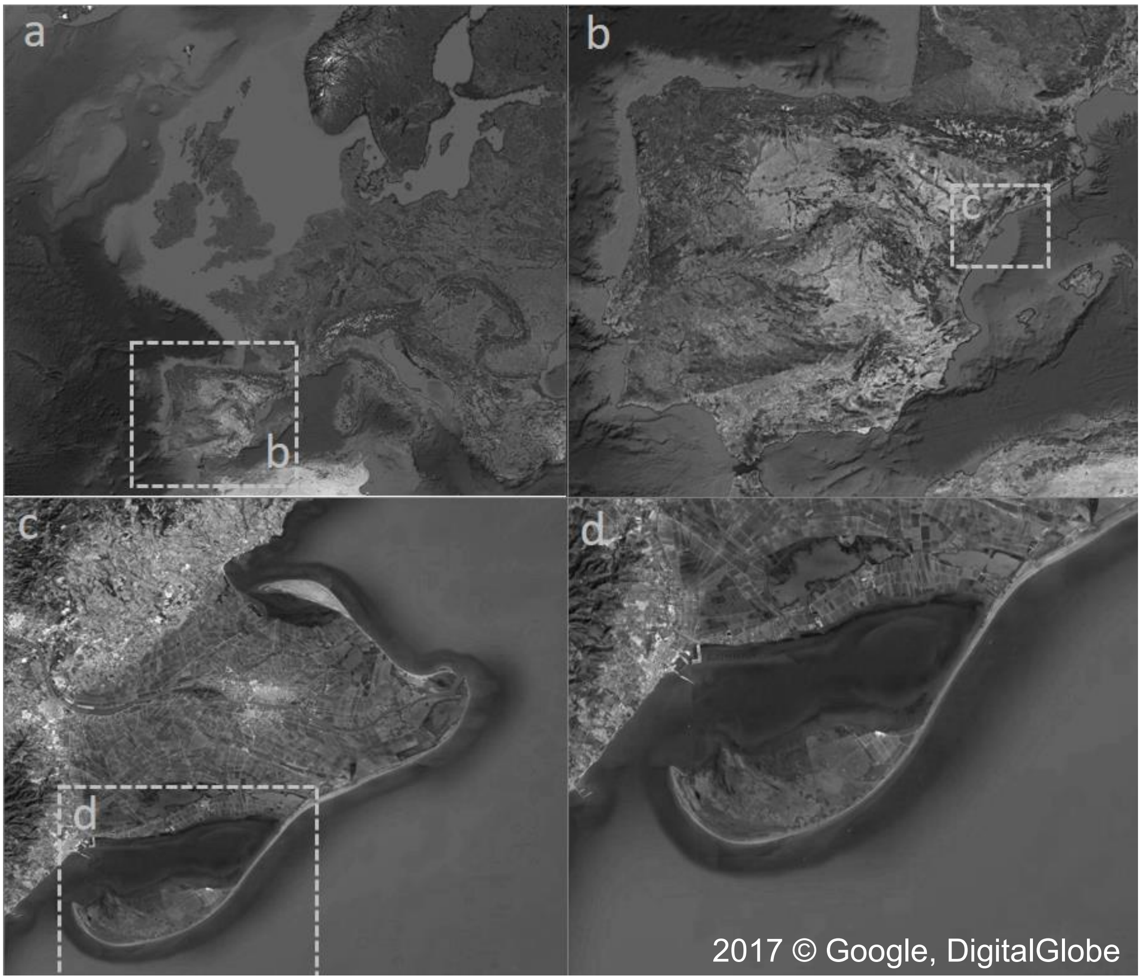

Figure 2. Location of the study area. Each panel zooms into the study area. Dashed line boxes represent the zoomed area in the next panel. a) Europe, b) Iberian Peninsula, c) Ebro Delta and d)breeding area in Ebro Delta. Maps copyright: 2017 © Google, DigitalGlobe.

Breeding performance

Parameters related to eggs are an excellent proxy of breeding conditions[1]. In the period 1992–2015 total number of nests in the Ebro Delta was counted and breeding performance was monitored annually. Transects covering the entire breeding area were conducted by several experienced researchers to count the number of nests (i.e. number of breeding pairs) and record their content (nests without eggs were not included). In particular, we recorded clutch size (the number of eggs laid) of all nests and measured egg length (L) and maximum egg width (W) with a digital calliper to the nearest (to the nearest ± 0.01mm) in a sample of 15 to 50 nests by patch (see [1]). Regular visits (median seven visits for the laying and incubation period lasting 30 days) allowed us to obtain unbiased estimates for clutch size. Egg volume (in cm^3) was calculated using the equation: $V= \beta (L)(W)^2$, in which β was a species-specific constant parameter (β = 0.476 for Audouin's gull [2,3]), L and W are expressed in cm. Egg volume and clutch size are often related to food availability [1,3]. To reduce the biases of food availability effects on egg volume we considered only egg volume of clutches with the mode clutch size by year. Moreover, we tested for potential correlations between predator intensity and food availability (tonnes of trawlers landed in the nearest fishing port) and found them to be independent.

Breeding success was estimated as the number of chicks (estimated using the Lincoln-Petersen estimator[4–6], see below) divided by the number of breeding pairs (i.e. nests) counted during the census. Chicks of Audouin's gull are nidifugous, they usually move and hide under vegetation, and consequently the only reliable methodology to estimate their number is by capture-mark-recapture methods (direct count is only possible for very small patches). Chicks were marked at c. 30 days old and the Lincoln-Petersen estimator is based on the assumption that if a proportion of the population was marked on a first occasion, after complete mixing, and a second sample was taken, then the proportion of marked individuals (chicks in the second sample would be the same as was marked initially in the total population. N=MT/R, N is population size, M is the number of individuals (chicks) marked on the first occasion, R is the number of marked chicks recaptured, T is the total number of individuals (chicks) captured on the second occasion. In order to use the Lincoln-Petersen estimator to estimate the number of chicks one team of researchers marked a sample of chicks in a patch (M), then a different team (often 1-3 days after) inspected the patch and counted the number of marked (R) and unmarked chicks (T-R).

References

1. Oro,D., O., L, J. & X, R. Influence of trawling activity on the breeding ecology of a threatened seabird, Audouin's gull Larus audouinii. *Mar. Ecol. Prog. Ser.* **139,** 19–29 (1996).
2. Harris, M. P. Aspects of the Breeding Biology of the Gulls. *Ibis* **106,** 432–456 (1964).
3. Payo-Payo, A. *et al.* Population control of overabundant species achieved through consecutive anthropogenic perturbations. *Press*
4. Oro, D. & Ruxton, G. D. The formation and growth of seabird colonies: Audouin's gull as a case study. *J. Anim. Ecol.* **70,** 527–535 (2001).
5. Seber, G. A. F. *The estimation of animal abundance*. (Blackburn press, 2002).
6. Williams, B. K., Nichols, J. D. & Conroy, J. M. *Analysis and Management of Animal Populations.* (Academic Press, 2001).
7. R Core Team. *R: A language and environment for statistical computing.* (R Foundation for Statistical Computing, 2013).
